# An Approximate Cone Beam Reconstruction Algorithm for Gantry-Tilted CT Using Tangential Filtering

**DOI:** 10.1155/IJBI/2006/29370

**Published:** 2006-06-01

**Authors:** Ming Yan, Cishen Zhang, Hongzhu Liang

**Affiliations:** ^1^ School of Electrical and Electronic Engineering, Nanyang Technological University, 639798, Singapore; ^2^ School of Chemical and Biomedical Engineering, Nanyang Technological University, 639798, Singapore

## Abstract

FDK algorithm is a well-known 3D (three-dimensional) approximate algorithm for CT (computed tomography) image reconstruction and is also known to suffer from considerable artifacts when the scanning cone angle is large. Recently, it has been improved by performing the ramp filtering along the tangential direction of the X-ray source helix for dealing with the large cone angle problem. In this paper, we present an FDK-type approximate reconstruction algorithm for gantry-tilted CT imaging. The proposed method improves the image reconstruction by filtering the projection data along a proper direction which is determined by CT parameters and gantry-tilted angle. As a result, the proposed algorithm for gantry-tilted CT reconstruction can provide more scanning flexibilities in clinical CT scanning and is efficient in computation. The performance of the proposed algorithm is evaluated with turbell clock phantom and thorax phantom and compared with FDK algorithm and a popular 2D (two-dimensional) approximate algorithm. The results show that the proposed algorithm can achieve better image quality for gantry-tilted CT image reconstruction.


Hindawi Publishing Corporation
International Journal of Biomedical Imaging
Volume 2006, Article ID 29370, Pages 1–8
DOI 10.1155/IJBI/2006/29370

### An Approximate Cone Beam Reconstruction Algorithm for Gantry-Tilted CT Using Tangential Filtering

**Ming Yan,[1] Cishen Zhang,[1,2] and Hongzhu Liang[1]**

[1] *School of Electrical and Electronic Engineering, Nanyang Technological University, Singapore 639798*
[2] *School of Chemical and Biomedical Engineering, Nanyang Technological University, Singapore 639798*

Received 30 November 2005; Revised 8 March 2006; Accepted 6 April 2006

FDK algorithm is a well-known 3D (three-dimensional) approximate algorithm for CT (computed tomography) image reconstruction and is also known to suffer from considerable artifacts when the scanning cone angle is large. Recently, it has been improved by performing the ramp filtering along the tangential direction of the X-ray source helix for dealing with the large cone angle problem. In this paper, we present an FDK-type approximate reconstruction algorithm for gantry-tilted CT imaging. The proposed method improves the image reconstruction by filtering the projection data along a proper direction which is determined by CT parameters and gantry-tilted angle. As a result, the proposed algorithm for gantry-tilted CT reconstruction can provide more scanning flexibilities in clinical CT scanning and is efficient in computation. The performance of the proposed algorithm is evaluated with turbell clock phantom and thorax phantom and compared with FDK algorithm and a popular 2D (two-dimensional) approximate algorithm. The results show that the proposed algorithm can achieve better image quality for gantry-tilted CT image reconstruction.

Copyright © 2006 Ming Yan et al. This is an open access article distributed under the Creative Commons Attribution License, which permits unrestricted use, distribution, and reproduction in any medium, provided the original work is properly cited.

### 1. INTRODUCTION

In some applications of clinical CT scanning, it is required that the CT gantry be tilted. For example, in order to avoid exposure of eyes to X-rays, gantry is tilted during the head scanning procedure. To meet such special requirements, a number of algorithms have been developed for gantry-tilted helical MSCT (multislice computed tomography) image reconstruction [1–4]. Among these algorithms, Kacherließ et al. [2] developed a gantry-tilted reconstruction algorithm based on the earlier developed 2D algorithm ASSR [5]. Hein et al. [3] developed a gantry-tilted reconstruction algorithm based on the 3D FDK algorithm [6, 7]. In the gantry-tilted FDK algorithm in [3], the reconstruction plane is perpendicular to the rotating axis of the CT scanning. Thus the scanning cone angle increases with the pitch value and slice number which can lead to unavoidable artifacts. Recently, Noo et al. [4] developed a general framework which can be applied to the gantry-tilted CT for exact [8, 9] and approximate image reconstruction.

The FDK-type algorithms for the approximate reconstruction using the projection data filtering in the horizontal direction suffer from considerable artifacts due to the large scanning cone angle for both normal and gantry-tilted helical MSCT imaging. Recently, some of the improvements have been made for the FDK-type algorithms. The tilted plane technique is combined with the FDK algorithm to reduce the cone angle and further reduce artifacts caused by the large cone angle [10]. Several methods reduce the artifacts by filtering projection data along the tangential direction of the helix [11–13]. Such a technique was earlier proposed by Yan and Leahy [11] and further improved by Sourbelle and Kalender [12] for short scan FDK-type algorithms. The improved FDK-type algorithm can achieve better image quality than that of the conventional FDK algorithm.

Motivated by the observation that artifacts for FDK-type algorithms can be effectively reduced by filtering projection data along the helix tangential direction, this paper extends the existing tangential filtering technique to present a 3D FDK-type approximation algorithm for gantry-tilted helical MSCT. Taking into account the gantry-tilted geometry, we provide a general formula for gantry-tilted CT reconstruction for different gantry-tilted angles. As a special case, the reconstruction formula reduces to the standard tangential filtering for conventional CT with zero gantry-tilted angle.

To deal with the complicated gantry-tilted geometry and large scanning cone angle, our proposed algorithm first

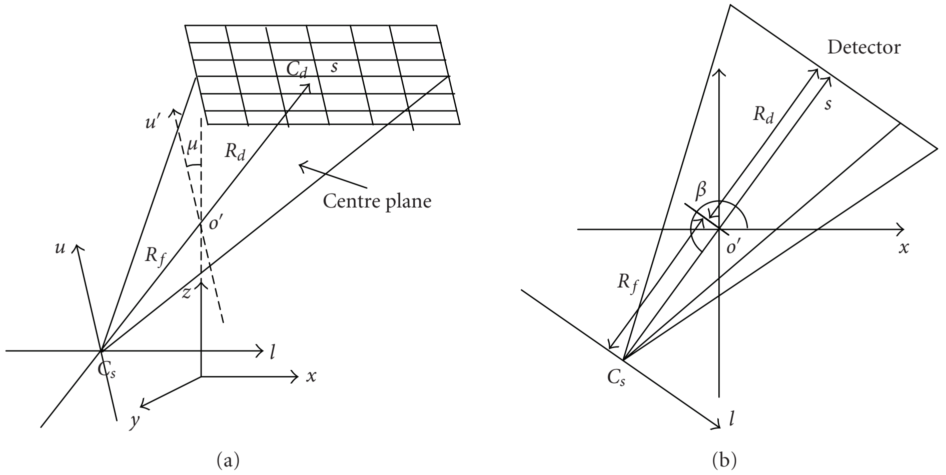

Figure 1: Geometry for gantry-tilted multislice CT scanner.

applies the ramp filtering to reconstruct a sequence of image planes and these planes are perpendicular to the rotating axis. The final horizontal image planes are then obtained by interpolating the tilted image planes. We present simulation results to show that the proposed gantry-tilted algorithm can provide considerably improved imaging for large scanning cone angle.

The key technique of the proposed algorithm is to reconstruct images on planes perpendicular to the rotation axis using the ramp filtering along the helix tangential direction. This is essentially different from the tilted plane reconstruction technique, such as that in [10], which is based on geometrically optimized reconstruction plane for reducing the effective cone angle.

It is noted that the tangential filtering technique can be implemented as a special case of the general framework for gantry-tilted CT proposed by Noo et al. [4]. Following its procedure, the gantry-tilted geometry is first transformed into a conventional CT scanning geometry with zero gantry-tilted angle and a projection data set of the transformed scanning geometry is formed. Then the tangential filtering technique can be applied on the data set for the image reconstruction. The procedure of the proposed algorithm in this paper is different from Noo et al. 's general framework in that it computes the helix tangential direction and applies the filtering directly without rebinning the projection data set for the transformed scanning geometry. This leads to more efficient computing and image reconstruction.

The rest of this paper is organized as follows. Section 2 presents the geometric scheme of the gantry-tilted CT. Section 3 is on the projection data set formation for tangential filtering. The proposed approximate reconstruction algorithm presented in Sections 4 and 5 is on simulation and evaluation of the proposed algorithm.

### 2. GEOMETRY FOR GANTRY-TILTED HELICAL MSCT

The helical MSCT scanning set up consists of an X-ray source and a detector array forming a source-detector framework. The geometry of the source-detector framework in a global cartesian coordinate system $x-y-z$ is shown in Figure 1(a), where $C_s$ denotes the X-ray source, the detector array is a rectangular surface with a geometric centre $C_d$. The source-detector framework defines a rotating cartesian coordinate system $s-l-u$ with the X-ray source $C_s$ as the origin, the straight line $C_s - C_d$ being the $s$ axis, the $l$ axis being parallel to the horizontal lines, and the $u$ axis being parallel to the vertical lines of the rectangular detector surface.

The $s-l$ plane containing $C_s$ and $C_d$ is called the centre plane and its geometry is shown in Figure 1(b), where $\beta$ is the projection angle, the distances from the rotating centre point $o'$ to the detector and the X-ray source are $R_d$ and $R_f$, respectively. In the gantry-tilted scanning process, the source-detector framework rotates around the axis $u'$ which is parallel to the $u$ axis and intersects the $z$ axis at $o'$ as shown in Figure 1(a). The gantry-tilted angle is represented by $\mu$ between the $z$ and $u'$ axes. And the projection angle $\beta$ is defined in the $s-l$ plane as shown in Figure 1(b).

For gantry-tilted CT, the X-ray source trajectory can be considered as a combination of two movements: the X-ray source rotates on the tilted circular trajectory with a radius $R_f$ and the centre of the circle moves straightforward along the table feeding direction. As a result, the X-ray source trajectory is the sum of the circular rotation trajectory on the centre plane and the linear translation in the $z$ direction in the following:

$$C_s(\beta) = \begin{pmatrix} x_s \\ y_s \\ z_s \end{pmatrix} = \begin{pmatrix} 1 & 0 & 0 \\ 0 & \cos\mu & -\sin\mu \\ 0 & \sin\mu & \cos\mu \end{pmatrix} \begin{pmatrix} R_f\cos\beta \\ R_f\sin\beta \\ 0 \end{pmatrix} + \begin{pmatrix} 0 \\ 0 \\ z_0 + \dfrac{pSM}{2\pi}(\beta-\beta_0) \end{pmatrix} = \begin{pmatrix} R_f\cos\beta \\ R_f\cos\mu\sin\beta \\ z_0 + \dfrac{pSM}{2\pi}(\beta-\beta_0) + R_f\sin\mu\sin\beta \end{pmatrix}, \quad (1)$$

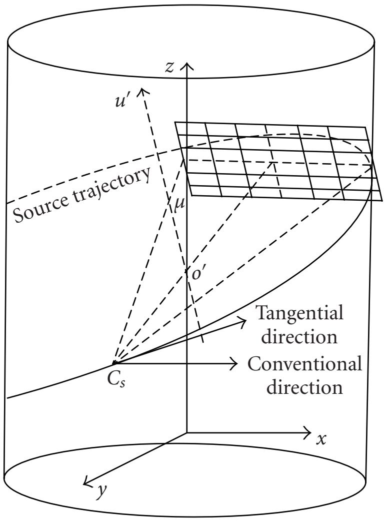

Figure 2: Illustration of conventional filter direction and tangential filter direction.

where $\beta_0$ is the initial projection angle, $z_0$ is the initial $z$ position of the centre of the gantry, $p$ is the pitch value of the helical cone beam scanning, $S$ is the slice thickness, and $M$ is the number of detector slice.

With the X-ray source trajectory given in (1), we take the derivative with respect to $\beta$ to obtain its tangential direction at $C_s$ in the global coordinate system represented in the following form:

$$\begin{pmatrix} x_t \\ y_t \\ z_t \end{pmatrix} = \frac{dC_s(\beta)}{d\beta} = \begin{pmatrix} -R_f \sin\beta \\ R_f \cos\mu \cos\beta \\ h + R_f \sin\mu \cos\beta \end{pmatrix}, \tag{2}$$

where $h = pSM/2\pi$. The X-ray source trajectory and its tangential direction at $C_s$ are shown in Figure 2.

It follows from the geometric relation between the global $x - y - z$ and the rotating $s - l - u$ coordinate system, as shown in Figures 1(a) and 1(b), that the transformation of the tangential direction $(x_t, y_t, z_t)^T$ as given in (2) to the rotating $s - l - u$ coordinate system can be obtained by first rotating this vector by an angle $\mu$ around the $x$ axis followed a rotation angle of $\beta - \pi$ around the $u$ axis. This results in the trajectory tangential direction in the rotating $s - l - u$ coordinate system, denoted by $(s_t, l_t, u_t)^T$, in the following:

$$\begin{aligned} \begin{pmatrix} s_t \\ l_t \\ u_t \end{pmatrix} &= \begin{pmatrix} \cos(\beta-\pi) & \sin(\beta-\pi) & 0 \\ -\sin(\beta-\pi) & \cos(\beta-\pi) & 0 \\ 0 & 0 & 1 \end{pmatrix} \\ &\quad \times \begin{pmatrix} 1 & 0 & 0 \\ 0 & \cos\mu & \sin\mu \\ 0 & -\sin\mu & \cos\mu \end{pmatrix} \begin{pmatrix} -R_f \sin\beta \\ R_f \cos\mu \cos\beta \\ h + R_f \sin\mu \cos\beta \end{pmatrix} \\ &= \begin{pmatrix} -h \sin\beta \sin\mu \\ R_f + h \sin\mu \cos\beta \\ h \cos\mu \end{pmatrix}. \end{aligned} \tag{3}$$

To implement the tangential filtering for the gantry-tilted scanning projection data, we further use the tangential direction $(s_t, l_t, u_t)^T$ to set up a tangential cartesian coordinate system $\xi - \eta - \zeta$. The origin of this coordinate system is the X-ray source $C_s$, the $\eta$ axis is defined by the tangential direction of the X-ray source trajectory at $C_s$, as given in (3), the $\zeta$ axis is parallel to the detector plane, and the $\xi - \zeta$ plane is orthogonal to the $\eta$ axis.

Using the tangential direction $(s_t, l_t, u_t)^T$ in (3), we introduce two angular quantities $\sigma$ and $\gamma$ as follows:

$$\begin{aligned} \sigma &= \arcsin\left(\frac{s_t}{\sqrt{s_t^2 + l_t^2}}\right) \\ &= \arcsin\left(\frac{-h \sin\mu \sin\beta}{\sqrt{R_f^2 + 2hR_f \sin\mu \cos\beta + h^2 \sin^2\mu}}\right), \end{aligned} \tag{4}$$

$$\begin{aligned} \gamma &= \arctan\left(\frac{u_t}{\sqrt{s_t^2 + l_t^2}}\right) \\ &= \arctan\left(\frac{h \cos\mu}{\sqrt{R_f^2 + 2hR_f \sin\mu \cos\beta + h^2 \sin^2\mu}}\right). \end{aligned} \tag{5}$$

Following from its definition, the $\xi - \eta - \zeta$ coordinate system can be obtained by first rotating the $s - l - u$ coordinate system around the $u$ axis by an angle $\sigma$ to obtain an intermediate coordinate system $\xi - \tilde{\eta} - u$ followed by rotating the $\xi - \tilde{\eta} - u$ coordinate system around the $\xi$ axis by an angle $\gamma$. Such coordinate rotations and transformation are illustrated in Figure 3. It follows that the transformation between the $s - l - u$ and $\xi - \eta - \zeta$ coordinate systems is given by

$$\begin{pmatrix} \xi \\ \eta \\ \zeta \end{pmatrix} = T \begin{pmatrix} s \\ t \\ u \end{pmatrix}, \tag{6}$$

where the transformation matrix $T$ is

$$\begin{aligned} T &= \begin{pmatrix} 1 & 0 & 0 \\ 0 & \cos\gamma & \sin\gamma \\ 0 & -\sin\gamma & \cos\gamma \end{pmatrix} \begin{pmatrix} \cos\sigma & \sin\sigma & 0 \\ -\sin\sigma & \cos\sigma & 0 \\ 0 & 0 & 1 \end{pmatrix} \\ &= \begin{pmatrix} \cos\sigma & \sin\sigma & 0 \\ -\sin\sigma \cos\gamma & \cos\sigma \cos\gamma & \sin\gamma \\ \sin\sigma \sin\gamma & -\cos\sigma \sin\gamma & \cos\gamma \end{pmatrix}. \end{aligned} \tag{7}$$

### 3. DATA REFORMATION FOR TANGENTIAL FILTERING

In conventional FDK-type algorithms, the ramp filtering is performed on detector rows and the filtering direction is parallel to the $l$ axis on the detector surface. Motivated by the tangential filtering technique for CT without gantry tilting, we propose in this paper that the ramp filtering is performed in the tangential direction of the X-ray source trajectory of the gantry-tilted CT as shown in Figure 2. For this purpose, the projection data are reformed such that rows of the reformed projection data set are parallel to the tangential direction of the X-ray source trajectory. The reformed projection

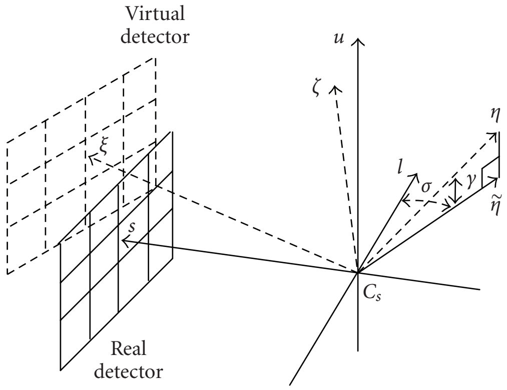

FIGURE 3: Illustration of rotation angles between $s{-}l{-}u$ and $\xi{-}\eta{-}\zeta$.

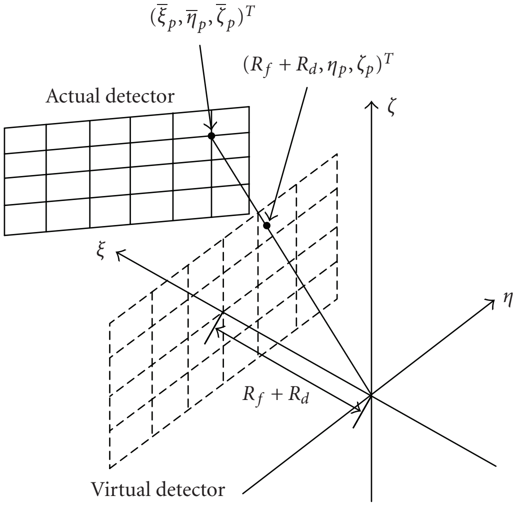

FIGURE 4: Illustration of the relationship between $(\bar{\zeta_p}, \bar{\eta_p}, \bar{\xi_p})$ and $(R_f + R_d, \eta_p, \zeta_p)$ in coordinate system $(\zeta, \eta, \xi)$.

data set enables the tangential filtering technique for the conventional CT reconstruction being applied.

The reformed data set is built up by introducing a virtual detector array surface in the tangential coordinate system $\xi - \eta - \zeta$ and transforming the projection data from the real detector array surface in the rotating $s - l - u$ coordinate system to the virtual detector array surface. The virtual detector array surface is placed on the plane $\xi = (R_f + R_d)$, as shown in Figure 4, with the rows being parallel to the $\eta$ axis.

As sown in Figure 4, each projection ray radiates on the real detector array at the point $((R_f + R_d), l_p, u_p)^T$ in the $s{-}l{-}u$ coordinate also radiates on a point $((R_f + R_d), \eta_p, \zeta_p)^T$ on the virtual detector array. Let the $\xi - \eta - \zeta$ coordinate of the point $((R_f + R_d), l_p, u_p)^T$ on the real detector array surface be $(\bar{\xi}_p, \bar{\eta}_p, \bar{\zeta}_p)^T$. The transformation from $((R_f + R_d), l_p, u_p)^T$ to $(\bar{\xi}_p, \bar{\eta}_p, \bar{\zeta}_p)^T$ is determined by the coordinate transformation matrix $T$ in (7), that is,

$$\begin{pmatrix} \bar{\xi}_p \\ \bar{\eta}_p \\ \bar{\zeta}_p \end{pmatrix} = T \begin{pmatrix} (R_f + R_d) \\ l_p \\ u_p \end{pmatrix}. \tag{8}$$

In view of Figure 4, the coordinate $(\eta_p, \zeta_p)^T$ of the virtual detector cell can be expressed, in terms of that of the real detector cell in the $\xi - \eta - \zeta$ coordinate system, as

$$\eta_p = \frac{R_f + R_d}{\bar{\xi}_p} \bar{\eta}_p, \qquad \zeta_p = \frac{R_f + R_d}{\bar{\xi}_p} \bar{\zeta}_p. \tag{9}$$

This, together with (8), can determine the coordinate of the virtual detector cell $(\eta_p, \zeta_p)^T$ in the tangential coordinate system $\xi - \eta - \zeta$ from a given real detector cell at $((R_f + R_d), l_p, u_p)^T$ in the rotating coordinate system.

Let the projection datum collected from the real detector cell at $((R_f + R_d), l_p, u_p)^T$ in the $s - l - u$ coordinate at the projection angle $\beta$ be denoted by $D(\beta, l_p, u_p)$ and the corresponding projection datum on the virtual detector cell at $((R_f + R_d), \eta_p, \zeta_p)^T$ in the $\xi - \eta - \zeta$ coordinate by the same projection ray be denoted by $D_v(\beta, \eta_p, \zeta_p)$. Since $D(\beta, l_p, u_p)$ and $D_v(\beta, \eta_p, \zeta_p)$ are due to the same projection ray, we have

$$D_v(\beta, \eta_p, \zeta_p) = D(\beta, l_p, u_p). \tag{10}$$

This can be used to obtain the reformed projection data set following from the coordinate transform from each real detector cell at $((R_f + R_d), l_p, u_p)^T$ in the rotating coordinate system to the corresponding virtual detector cell at $((R_f + R_d), \eta_p, \zeta_p)^T$ in the tangential coordinate system.

### 4. RECONSTRUCTION AND INTERPOLATION

Because the gantry is tilted and final images should be on horizontal planes in the global $x - y - z$ coordinate system, we first reconstruct images on a sequences of intermediate planes parallel to the centre plane and then interpolate them to obtain the horizontal images. The introduction of the intermediate planes effectively reduces the projection cone angle in comparison with constructing directly the horizontal planes. Let $\psi = \{P_i : i = 1, 2, 3, \ldots, n\}$ be the set of the intermediate planes and let the intersection of each tilted plane $P_i$ and the $z$-axis be at point $o_i = (0, 0, z_i)^T$, with $z_i = z_0 + i\Delta z$, $i = 1, 2, 3, \ldots, n$, and $\Delta z > 0$. The projection angle for the X-ray source being at $z_i$ in the $z$ direction is

$$\beta_i = \beta_0 + \frac{2\pi i \Delta z}{pSM}. \tag{11}$$

And the equation for the intermediate plane $P_i$ is

$$z = z_i + y \tan \mu. \tag{12}$$

The ramp filtering is along the $\eta$ direction which is defined as the tangential direction of the source trajectory. Let $g(\cdot)$ denote the ramp filter. Applying it to the reformed data set on the virtual detector array in the tangential direction of the source trajectory and performing standard cone beam weighting yields

$$\widetilde{D}(\beta, \eta', \zeta) = \int \frac{R_f D_v(\beta, \eta, \zeta)}{\sqrt{\eta'^2 + R_f^2 + \zeta^2}} g(\eta' - \eta) d\eta. \tag{13}$$

Table 1: Parameters of the clock phantom (mm).

| No. | Centre | No. | Centre | No. | Centre | No. | Centre |
|---|---|---|---|---|---|---|---|
| 1 | (0,200,0) | 7 | (0,−200,−12) | 13 | (0,100,0) | 19 | (0,−100,−12) |
| 2 | (100,173.2,−2) | 8 | (−100,−173.2,−14) | 14 | (50,86.6,−2) | 20 | (−50,−86.6,−14) |
| 3 | (173.2,100,−4) | 9 | (−173.2,−100,−16) | 15 | (86.6,50,−4) | 21 | (−86.6,−50,−16) |
| 4 | (200,0,−6) | 10 | (−200,0,−18) | 16 | (100,0,−6) | 22 | (−100,0,−18)) |
| 5 | (173.2,−100,−8) | 11 | (−173.2,100,−20) | 17 | (86.6,−50,−8) | 23 | (−86.6,50,−20) |
| 6 | (100,−173.2,−10) | 12 | (−100,173.2,−22) | 18 | (50,−86.6,−10) | 24 | (−50,86.6,−22) |

Then the reconstruction formula is derived from Yan and Leahy's [11] paper for tangential filtering reconstruction.

$$f_i(x,y,z) = \frac{1}{2}\int_{\beta_i-\beta_m/2}^{\beta_i+\beta_m/2} \frac{R_f \| dC_s(\beta)/d\beta \| \widetilde{D}(\beta,\eta'(\beta),\zeta(\beta))}{(x\cos\beta + y\sin\beta - R_f)^2} d\beta = \frac{1}{2}\int_{\beta_i-\beta_m/2}^{\beta_i+\beta_m/2} \frac{R_f\sqrt{R_f^2+2hR_f\sin\mu\cos\beta+h^2}\widetilde{D}(\beta,\eta'(\beta),\zeta(\beta))}{\left(x\cos\beta + y\sin\beta - R_f\right)^2} d\beta, \quad (14)$$

where $\|\cdot\|$ denotes Euclidean norm and $\beta_m$ is the the length of the projection angle interval.

Given the reconstructed images of the tilted planes $P_i$, for $i = 1,2,3,\ldots,n$, and a point $(x,y,z)^T$ on a plane parallel to the $x-y$ plane where the image is to be obtained by interpolation, the $z$-positions of the intermediate planes $P_i$, $i = 1,2,3,\ldots,n$, at $(x,y)^T$ and denoted by $z_{p_i}(x,y)$ can be determined by (12). Thus there exist two points $(x,y,z_{P_j})^T$ and $(x,y,z_{P_k})^T$ on two tilted planes $P_j$ and $P_k$, respectively, at the upper and lower sides of $(x,y,z)^T$, respectively, which are closest to $(x,y,z)^T$. Using the obtained attenuation functions $f_j(x,y,z_{P_j})$ and $f_k(x,y,z_{P_k})$ for planes $P_j$ and $P_k$, respectively, the interpolated attenuation function can be obtained using the following interpolation formula:

$$f(x,y,z) = \frac{f_j(x,y,z_{P_j}(x,y))(z_{P_k}-z)}{z_{P_k}-z_{P_j}} + \frac{f_k(x,y,z_{P_k}(x,y))(z-z_{P_j})}{z_{P_k}-z_{P_j}}. \quad (15)$$

We can now summarise the proposed reconstruction procedure in the following.

(1) Determine the $z$ position $z_i$, centre of the optimal plane $P_i$, its centre projection angle $\beta_i$, and obtain the reconstruction plane using (12). For half-scan, $\beta_m = \pi + \theta_{\text{fan}}$.
(2) Obtain the reformed projection data set on the virtual detector array using (9).
(3) Reconstruct the image on the intermediate planes $P_i$ using (14).
(4) Obtain the horizontal images by interpolating the intermediate images.

### 5. SIMULATION

We use Turbell clock phantom and thorax phantom to evaluate the performance of the proposed algorithm. Parameters of the simulation are set to $R_f = 570$ mm, $R_d = 560$ mm, $S = 1$ mm, $p = 1.0$, projection number per rotation $N_p = 1024$, number of detector cells for each row $N_f = 800$, detector cell width 1.5 mm, gantry-tilted angle $\mu = 10^\circ$. The simulated performance of the proposed algorithm is compared with two existing approximate gantry-tilted CT reconstruction algorithms, which are the gantry-tilted FDK algorithm in [3] by Hein et al. in 2003 and the gantry-tilted ASSR [2] by Kachelrei$\beta$ et al in 2001.

The Turbell clock phantom is used to evaluate the proposed algorithm. The Turbell clock phantom is composed with a cylinder and two group balls. The radius of the cylinder is 240 mm and its centre is (0,0,0) and its length is 100 mm and its value is 0.4. The radius of the outer group balls is 22 mm and their values are 1.0. The radius of inner group balls is 12 mm and their values are 1.0. The positions of these balls are listed in Table 1. Results obtained from the simulation of Turbell clock phantom are shown in Figure 5, the left column is reconstructed images and the right column is the centre vertical line for reconstructed images. In Figure 5, Figure 5(a) is the image reconstructed by our proposed algorithm, Figure 5(c) is reconstructed by the gantry-tilted FDK algorithm and Figure 5(e) shows the image reconstructed by the gantry-tilted ASSR. The results demonstrate that the image reconstructed by our proposed algorithm contains fewer artifacts than that of the other two existing algorithms for gantry-tilted CT.

In the simulation of Turbell clock phantom, the centre vertical lines ($y = 0$ mm) for the reconstructed images are also constructed and displayed by the proposed algorithm (Figure 5(b)), gantry-tilted FDK algorithm (Figure 5(d)), and gantry-tilted ASSR (Figure 5(f)), repectively. In Figure 5, dashed lines represent the original image and solid lines represent reconstructed profiles. The results show that the profile of the proposed algorithm as shown in Figure 5(b) has smaller variance than the other two results.

A thorax phantom is further simulated which is designed by referring to human thorax consisting of many important organs and often scanned in CT examination. These organs include lungs, heart, aorta, ribs, spine, sternum, and shoulders. The phantom definitions are obtained from a

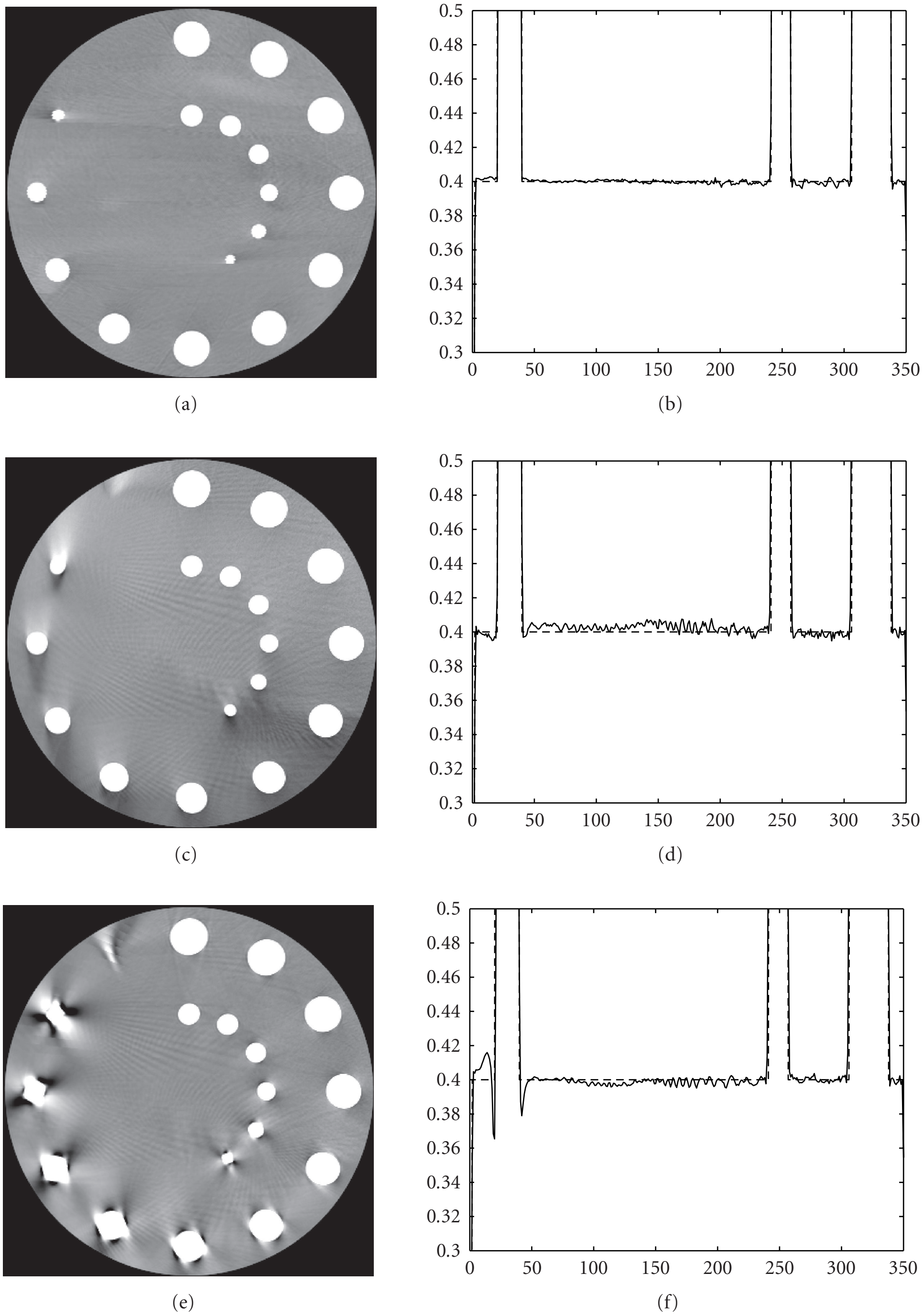

Figure 5: Images for the plane $z = 0$ mm of clock phantom generated with the proposed algorithm, gantry-tilted FDK, and gantry-tilted ASSR with $S = 1$ mm, $p = 1.0$, $M = 96$ $(0.35 - 0.45)$.

world phantom database FORBILD. The simulation results are shown in Figure 6, where Figure 6(a) is the original thorax phantom, Figure 6(b) shows the image reconstructed by the proposed algorithm, Figure 6(c) is the image reconstructed by the gantry-tilted FDK algorithm, and Figure 6(d) is reconstructed by the gantry-tilted ASSR algorithm. The right-hand column shows zoomed reconstruction images. It is shown that there are obvious artifacts around ribs in the images reconstructed by conventional FDK algorithm and ASSR algorithm. In contrast, the proposed algorithm provides better image quality and more accurate reconstruction.

### 6. CONCLUSION

This paper presents an approximate algorithm for gantry-tilted helical MSCT image reconstruction. It is based on

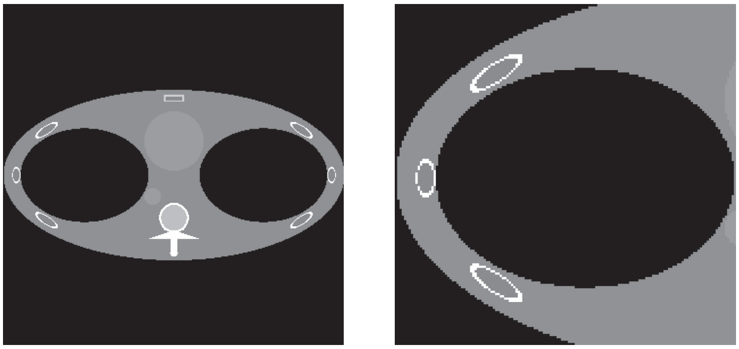

(a)

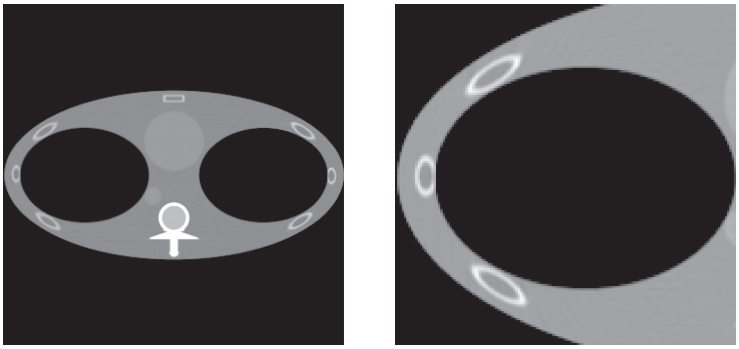

(b)

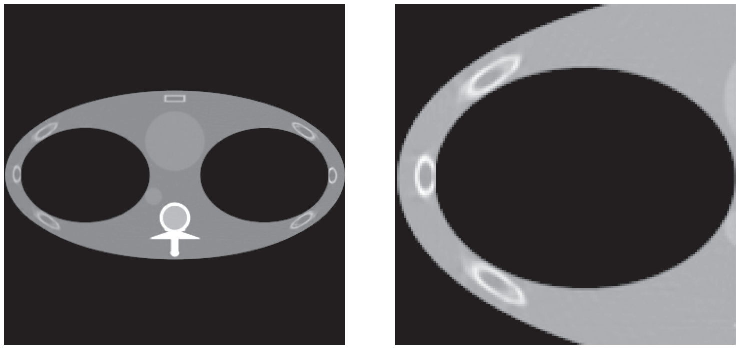

(c)

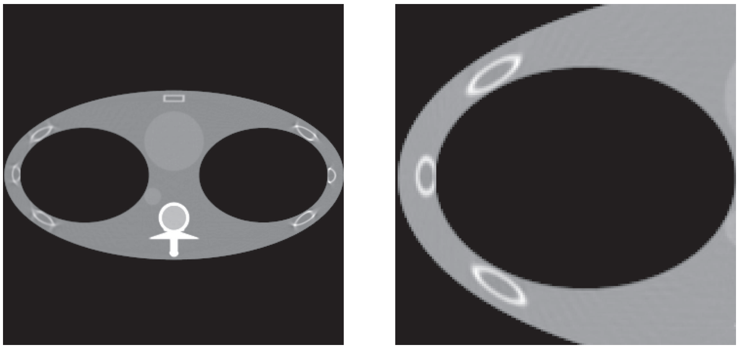

(d)

Figure 6: Images for the plane $z = 0$ mm of thorax phantom generated with the proposed algorithm, gantry-tilted FDK algorithm, and gantry-tilted ASSR algorithm with $M = 96$ (0.62-1.212).

the idea of filtering the 3D projection data and the filtering direction is varying and dependent on the CT parameters and the gantry-tilted angle. As a result, the proposed gantry-tilted reconstruction algorithm can provide more scanning flexibility in clinical CT scanning and is efficient in computation. In comparison with the existing 2D and 3D algorithms for gantry-tilted CT image reconstruction, our proposed algorithm can provide improved image quality. The performance of the proposed algorithm is evaluated with Turbell clock phantom and thorax phantom in comparison with the recent gantry-tilted FDK algorithm and gantry-tilted ASSR algorithm. The improved performance and image quality of the proposed algorithm have been shown.

### REFERENCES

[1] J. Hsieh, "Tomographic reconstruction for tilted helical multislice CT," *IEEE Transactions on Medical Imaging*, vol. 19, no. 9, pp. 864–872, 2000.

[2] M. Kachelrieß, T. Fuchs, S. Schaller, and W. A. Kalender, "Advanced single-slice rebinning for tilted spiral cone-beam CT," *Medical Physics*, vol. 28, no. 6, pp. 1033–1041, 2001.

[3] I. Hein, K. Taguchi, M. D. Silver, M. Kazama, and I. Mori, "Feldkamp-based cone-beam reconstruction for gantry-tilted helical multislice CT," *Medical Physics*, vol. 30, no. 12, pp. 3233–3242, 2003.

[4] F. Noo, M. Defrise, and H. Kudo, "General reconstruction theory for multislice X-ray computed tomography with a gantry tilt," *IEEE Transactions on Medical Imaging*, vol. 23, no. 9, pp. 1109–1116, 2004.

[5] M. Kachelrieß, S. Schaller, and W. A. Kalender, "Advanced single-slice rebinning in cone-beam spiral CT," *Medical Physics*, vol. 27, no. 4, pp. 754–772, 2000.

[6] I. A. Feldkamp, L. C. Davis, and J. W. Kress, "Practical cone-beam algorithm," *Journal of the Optical Society of America A*, vol. 1, no. 6, pp. 612–619, 1984.

[7] G. Wang, T.-H. Lin, P.-C. Cheng, and D. M. Shinozaki, "General cone-beam reconstruction algorithm," *IEEE Transactions on Medical Imaging*, vol. 12, no. 3, pp. 486–496, 1993.

[8] A. I. Katsevich, "Theoretically exact filtered backprojection-type inversion algorithm for spiral CT," *SIAM Journal on Applied Mathematics*, vol. 62, no. 6, pp. 2012–2026, 2002.

[9] P. Grangeat, "Mathematical framework of cone beam 3D reconstruction via the first derivative of the Radon transform," in *Mathematical Methods in Tomography*, vol. 1497 of *Lecture Notes in Mathematics*, pp. 66–97, Springer, Berlin, Germany, 1992.

[10] M. Yan and C. Zhang, "Tilted plane feldkamp type reconstruction algorithm for spiral cone beam CT," *Medical Physics*, vol. 32, no. 11, pp. 3455–3467, 2005.

[11] X. Yan and R. M. Leahy, "Cone beam tomography with circular, elliptical and spiral orbits," *Physics in Medicine and Biology*, vol. 37, no. 3, pp. 493–506, 1992.

[12] K. Sourbelle and W. A. Kalender, "Generalisation of Feldkamp reconstruction for clinical spiral cone beam CT," in *Proceedings of the 7th International Conference on Fully 3D Reconstruction in Radiology and Nuclear Medicine*, Saint-Malo, France, June-July 2003.

[13] M. Kachelrieß, M. Knaup, and W. A. Kalender, "Extended parallel backprojection for standard three-dimensional and phase-correlated four-dimensional axial and spiral cone-beam CT with arbitrary pitch, arbitrary cone-angle, and 100% dose usage," *Medical Physics*, vol. 31, no. 6, pp. 1623–1641, 2004.

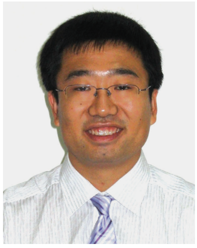

**Ming Yan** received the B.Eng. degree and the M.S. degree in automation, both from Tsinghua University, China, in 2000 and 2003, respectively. Since 2003, he has been pursuing the Ph.D. degree at the Nanyang Technological University, Singapore. His research interests include computed tomography reconstruction algorithms and medical imaging.

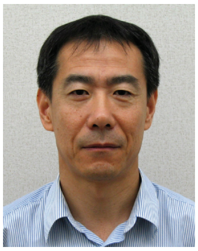

**Cishen Zhang** received the B.Eng. degree from Tsinghua University, China, in 1982 and the Ph.D. degree in electrical engineering from Newcastle University, Australia, in 1990. Between 1971 and 1978, he was an Electrician with Changxindian (February Seven) Locomotive manufactory, Beijing, China. He carried out research work on control systems at Delft University of Technology, The Netherlands, from 1983 to 1985. After his Ph.D. study from 1986 to 1989 at Newcastle University, he was with the Department of Electrical and Electronic Engineering at the University of Melbourne, Australia, as a Lecturer, Senior Lecturer, and Associate Professor and Reader till October 2002. He is currently with the School of Electrical and Electronic Engineering and School of Chemical and Biomedical Engineering at Nanyang Technological University, Singapore. His research interests include signal processing, medical imaging and control.

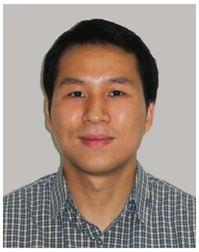

**Hongzhu Liang** received the B.Eng. degree in Xi'an Jiaotong University, China, in 2000, and the M.Eng. degree in Huazhong University of Science and Technology, China, in 2003. Since 2003 he has been with School of Electrical and Electronic Engineering, Nanyang Technological University, Singapore, as a research student pursuing the Ph.D. degree.

